# Compliance and Effectiveness of WHO Surgical Safety Check list: A JPMC Audit

**DOI:** 10.12669/pjms.324.9884

**Published:** 2016

**Authors:** Mariyah Anwer, Shahneela Manzoor, Nadeem Muneer, Shamim Qureshi

**Affiliations:** 1 Dr. Mariyah Anwer, Senior Registrar, Ward 2, Jinnah Postgraduate Medical Centre, Karachi, Pakistan; 2 Dr. Shahneela Manzoor, Postgraduate Trainee, Ward 2, Jinnah Postgraduate Medical Centre, Karachi, Pakistan; 3 Dr. Nadeem Muneer, Incharge, Department of Anesthesia, Jinnah Postgraduate Medical Centre, Karachi, Pakistan; 4 Prof. Shamim Qureshi, Head of the Department, Ward 2, Jinnah Postgraduate Medical Centre, Karachi, Pakistan

**Keywords:** WHO Surgical safety checklist, Safe Surgery Saves Lives initiative

## Abstract

**Objective:**

To assess World Health Organization (WHO) Surgical Safety Checklist (SSC), compliance and its effectiveness in reducing complications and final outcome of patients.

**Methods:**

This was a prospective study done in Department of General Surgery (Ward 02), Jinnah Postgraduate Medical Centre (JPMC), Karachi. The study included Total 3638 patients who underwent surgical procedure in elective theatre in four years from November 2011 to October 2015 since the SSC was included as part of history sheets in ward. Files were checked to confirm the compliance with regards to filling the three stage checklist properly and complications were noted.

**Results:**

In 1st year, out of 840 surgical procedures, SSC was properly marked in 172 (20.4%) cases. In 2nd year, out of 857 surgical procedures 303 (35.3%) cases were marked which increased in 3rd year out of 935 surgical procedures 757 (80.9%) cases and in 4th year out of 932 surgical procedures 838 (89.9%) cases were marked. No significant change in site and side (left or right) complications were noted in all four years. Surgical Site Infection (SSI) was noted in 59 (7.50%), 52 (6.47%), 44 (4.70%) and 20 (2.12%) cases in 1st, 2nd, 3rd and 4th year respectively. SSI in laparoscopic cholecystectomies was 41 (20.8 %), 45 (13%), 20 (5.68%) and 4 (1.12%) in 1st, 2nd, 3rd and 4th year respectively. No significant change in chest complications were noted in all four years. Mortality rate also remained same in all four years.

**Conclusion:**

WHO SSC is an effective tool in reducing in-hospital complications thus producing a favorable outcome. Realization its efficacy would improve compliance.

## INTRODUCTION

Hospitals are not as safe as generally believed.^1^ Surgical morbidity and mortality are rightly considered public health concerns. It has been estimated that more than 200 hundred million major surgical procedures are performed annually worldwide.^2^ Overall, the incidence of in-hospital adverse events is about 10 per cent, of which three-quarters are related to surgery. At least half of these adverse events are considered preventable within the current standards of care.^3^-^5^ Substantial improvements can be achieved by reducing variation in the reliability of surgical care processes.^6^ Briefings in the operating room improve team cooperation, motivation, discipline, and outcomes.^7^

With the aim of improving patient safety following surgery, a checklist was developed by the WHO patient safety programme. The SSC consists of 19 items and is used at three critical perioperative moments: induction, incision and before the patient leaves the operating theatre.^8^

Previous studies^9^-^11^ suggested that implementation compliance was low, despite checklist awareness by the theatre team. The knowledge that checklists are executed incompletely makes the evaluation of a team’s compliance with the checklist as important as evaluating clinical outcomes.^10^ The most common barrier was resistance or noncompliance from individual members of OR team (particularly at Attending level), which in many cases prevented the checklist from being used in the manner it was intended.^11^ Studies have demonstrated significant reduction in surgical morbidity and mortality after implementation of SSC during and /or after surgery.^12^,^13^

The aim of this study was to assess WHO SSC, compliance and its effectiveness in reducing complications and final outcome of patients in elective setting in public hospital.

## METHODS

It was a prospective study performed in General Surgery Ward-2, JPMC, Karachi, in four years from November 2011 to October 2015. A total of 3638 patients were enrolled in this study, underwent surgical procedure in elective theatre. WHO surgical safety checklist was made part of the ward file and was filled with each elective surgery. Surgical team, anesthetists and nurses were educated by presentations on how to fill it and who will fill it. Starting with sign in before induction when patient was shifted to O.T, Time out before skin incision and sign out after finishing operation was filled with each elective procedure. Files were later checked to confirm the compliance with regards to filling the three stage checklist properly.

Inclusion criteria was all elective list cases. Patients admitted in surgical ward from surgical OPD were enrolled in the study after taking informed consent. Consecutive sampling technique was used. We follow the patients for 30 days to record wound infection, chest complication and mortality.

Statistical analysis for students t-test was performed using SPSS (version 20.0). A p-value less than 0.05 was considered statistically significant. The study was approved by the JPMC Ethics Committee.

## RESULTS

In 1st year, out of 840 surgical procedures, Surgical safety checklist was properly marked in 172 (20.4%) cases which increased 838 (89.9%) cases in the fourth year. Table-I were marked. We took mean of first two years compliance and compared it to mean of last two years and applied t-test which showed p-value of less than 0.0001 which was statistically extremely significant. Fig.I

**Table-I:** Compliance of SSC during four years period.

No. of Years	No. of Elective procedures	SSC marked	Percentage
1^st^ Year	840	172	20.4%
2^nd^ Year	857	303	35.3%
3^rd^ Year	935	757	80.9%
4^th^ Year	932	838	89.9%

**Fig.1:**
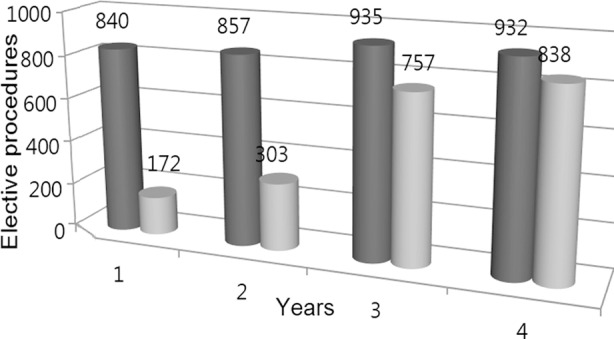
Number of elective procedures comparing SSC marked in four years.

No site and side (left or right) complications noted in all 4 years.

Surgical Site Infection (SSI) was noted in 59 (7.50%), 52 (6.47%) in the first year which reduced to 20(2.12%) in the fourth year. Table-II. SSI in laparoscopic cholecystectomies was 41 (20.8 %), 45 (13%), 20 (5.68%) and 4 (1.12%) in 1st, 2nd, 3rd and 4th year respectively. There was significant improvement in SSI incidence as seen by p-value of less than 0.0001 between first two and last two years. Fig.II

**Table-II:** SSI noted in all elective procedures and port site infection noted in laparoscopic cholecystectomy.

# of years	Total # of patients	SSI	Percentage	Laparoscopic cholecystectomy	Port site infection	Percentage
1st	840	59	7.50%	197	41	20.8%
2nd	857	52	6.06%	350	45	13%
3rd	935	44	4.70%	352	20	5.68%
4th	932	20	2.12%	355	4	1.12%

**Fig.2:**
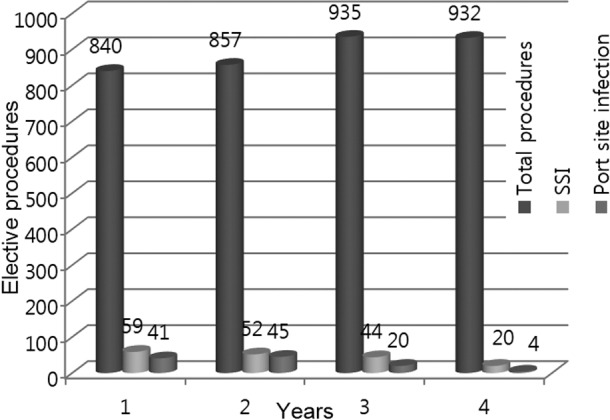
Yearly comparison of SSI and Port site infection in Elective procedures.

**Figure:**
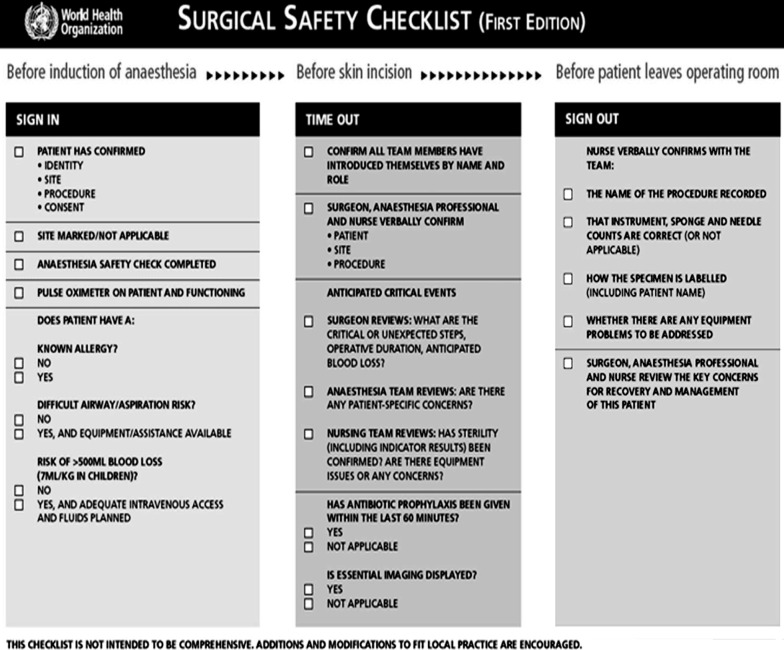


No chest complications noted in all four years. No site or side (right/left) error was noted. Mortality rate also remained same in all four years. (P-value of 1.00)

## DISCUSSION

Our study suggest that due to focus on improving team dynamics and communication there was a gradual increase seen in compliance to filling the SSC. Introducing the WHO SSC to the clinical environment can be challenging. The checklist is intended to give teams a simple, efficient set of priority checks for improving effective teamwork and communication and to encourage active consideration of the safety of patients in every operation performed. The checklist has two purposes: ensuring consistency in patient safety and introducing a culture that values achieving it. Experience shows that with education, practice and leadership, barriers to implementation can be overcome. With proper planning and commitment the checklist steps are easily accomplished and can make a profound difference in the safety of surgical care.^14^ The evaluation of a team’s compliance with the checklist, which is measured by adherence, is as important as evaluating outcomes.^10^,^11^ The efficacy of the checklist was found to be correlated with correct performance of the briefing.^15^ A retrospective study revealed that the use of the WHO checklist could have prevented 14.9% of all wrong-side errors) such as marking the wrong side).^16^

Faulty implementation can foster a dangerous false sense of security and thus convert the positive effect of checklist into its opposite. Therefore implementation process was time taken to enlist local leaders, educate staff in the benefits of adopting the Checklist, deliver formal training, and repeatedly reinforce Checklist use during the initial phase.

When we started filling the surgical safety checklist initially the compliance was low because of loyalty, fidelity and lack of commitment in some cases, inconsistent use of check list, team introductions were never performed but boxes were always ticked, team work was undetermined by staff being distracted, dismissive or absent during checks. Checkboxes were often ticked without obtaining the information and the timing of checks was not correct. Sometimes during procedures the nurse ticked the Sign In and Time Out checkboxes. This meant that equipment counts were ticked as complete when the equipment was still in use, and specimens were recorded as correctly labelled before they had been removed from the patient. Most often Time Out checkboxes were missed at the end of procedure which shows lack of completeness.

It has been suggested that the use of the WHO Checklist is associated with the development of a better safety attitude among the operating personnel.^17^ Checklists help surgeons to avoid making simple mistakes, such as surgery at wrong site. Despite more than a decade of campaigns by major organizations to prevent such events from occurring, there are still reports of such mistakes.^18^ In our study no site or side (right/left) error was noted.

In some studies Improving antibiotic delivery and timing has been shown to independently decrease rates of surgical site infection by 50% or more.^19^,^20^ Significant decrease in SSI rates following SSC implementation from 6.2% to 3.4%^13^, 11.2% to 6.6%^21^ and 14.9% to 4.7%^22^ have been reported.

After implementation of WHO SSC its effectiveness was noted in reduction of complications including surgical site wound infection and chest complications. Reduction in surgical site wound infection was remarkable after laparoscopic cholecystectomies due to making sure of application of pulse oximeter, administration of antibiotic and use of sterility indicators.

Chest complications, pneumonia or lower respiratory tract infections were reported in five studies.^13^,^23^,^24^ One^21^ reported a significant decrease in pneumonia rates. In our study no chest complication was noted. Mortality rates were relatively low, some studies were underpowered and as such not able to detect a potential difference in mortality.^21^-^24^ A recent meta-analysis found that the use of the WHO SSC improves patient safety in the operating room by decreasing postoperative complications and mortality.^25^ In our study no change in mortality rate was noted. Over all, our study suggests that higher adherence to filling of Surgical Safety Checklist can decrease complications hence proving it’s efficacy.

### Limitations of the study

It included only elective cases while emergency cases and short term complications were excluded.

## CONCLUSION

WHO SSC is an effective tool in reducing in-hospital complications thus producing a favorable outcome. Realization its efficacy would improve compliance.
